# Soil salinity and matric potential interaction on water use, water use efficiency and yield response factor of bean and wheat

**DOI:** 10.1038/s41598-018-20968-z

**Published:** 2018-02-08

**Authors:** Mahnaz Khataar, Mohammad Hossien Mohammadi, Farzin Shabani

**Affiliations:** 1 Department of Soil Science, University of Zanjan, Zanjan, Iran; 2 Department of Soil Science Faculty of Agriculture and Natural Resources, University of Tehran, Karaj, Iran; 3 Ecosystem Management, School of Environmental and Rural Science, University of New England, Armidale, NSW 2351 Australia

## Abstract

We studied the effects of soil matric potential and salinity on the water use (WU), water use efficiency (WUE) and yield response factor (Ky), for wheat (*Triticum aestivum* cv. Mahdavi) and bean (*Phaseoulus vulgaris* cv. COS16) in sandy loam and clay loam soils under greenhouse conditions. Results showed that aeration porosity is the predominant factor controlling WU, WUE, Ky and shoot biomass (Bs) at high soil water potentials. As matric potential was decreased, soil aeration improved, with Bs, WU and Ky reaching maximum value at −6 to −10 kPa, under all salinities. Wheat WUE remained almost unchanged by reduction of matric potential under low salinities (EC ≤ 8 dSm^−1^), but increased under higher salinities (EC ≥ 8 dSm^−1^), as did bean WUE at all salinities, as matric potential decreased to −33 kPa. Wheat WUE exceeds that of bean in both sandy loam and clay loam soils. WUE of both plants increased with higher shoot/root ratio and a high correlation coefficient exists between them. Results showed that salinity decreases all parameters, particularly at high potentials (h = −2 kPa), and amplifies the effects of waterlogging. Further, we observed a strong relationship between transpiration (T) and root respiration (Rr) for all experiments.

## Introduction

Soil aeration is an important factor affecting water uptake and growth in plants^1^. In wet soils (h > −10 to −30 kPa) aeration is limited, gas diffusion and transfer are insufficient, plants respiration is impeded and consequently water not absorbed efficiently^2^. As the frequency and connectivity of air filled pores increase, so does the soil matric suction, intensifying the uptake of water^2^. Oxygen deficiency symptoms occur below the critical value of air content, depending on plant and soil types. Thus, under flood irrigation, plants tolerant to lack of aeration such as the tomato, and a variety of forage species, commence water uptake within a few hours of irrigation, while species sensitive to aeration, such as the pea and potato, may take a few days, particularly in fine-textured soils^1^.

In addition to soil water content, soil salinity also influences the soil available water range, and subsequently the water use of plants. Salinity, via reduction of soil water potential and ionic toxicity, dramatically reduces the photosynthetic rate, transpiration rate (T), stomata conductance and root hydraulic conductance. Thus, plant water uptake and productivity decrease significantly^3^.

Plant productivity is dependent on the availability of soil water, in terms of both quantity and quality, as well as on water use efficiency (WUE = yield/water use)^4^. WUE is an important subject in agriculture, due to the increase in areas under irrigation and the high water requirements of crops, which consume around 70% of water available to humans^5^. WUE is considered an expression factor of yield value under stress conditions and can illustrate crop tolerance to drought stress. The yield production level is highly dependent on the transpiration rate. Effective use of water improves soil water uptake and subsequently transpiration rate and crop yield, so WUE significantly augments yield^6^. Increased water use efficiency can improve productivity and reduce stresses caused by environmental conditions^7^. This parameter is an expression of the relationship between crop yield and water use (evapotranspiration) during a growing season and is critically affected by soil available water and its limiting parameters^8^. In saturated and near-saturated soils, due to reduction of the oxygenation parameters such as air permeability, gas diffusion coefficient and air filled porosity^1^, the water uptake by plant roots decreases, leading to significant reductions in yield and WUE^9^. The association between WUE and soil moisture has been well researched for many crops, under different production systems, especially in waterlogged soils^10^. Dasila *et al*.^11^ showed that WUE in flooded irrigation conditions is very low, due to serious water and nutrient losses. A further complication is that an excess of soil water may not immediately exit the system, but may remain in the B horizon of texture-contrast soils as a perched water table^12^. This can lead to transient anoxia, stomata closing, yield and WUE reduction, and eventually root death, depending on the duration of waterlogging^12^. Létourneau *et al*.^13^ revealed that the best yield and WUE are obtained with a soil matric potential (based on irrigation thresholds) of −8 kPa in silty clay loam soil, or −10 to −15 kPa in clay loam soil, and suggested that WUE could be further improved by implementing high frequency irrigation.

Soil water salinity also affects WUE due to the ion specific toxicity and the decrease in available water, as well as the photosynthetic activity and crop yield. Semiz *et al*.^14^ stated that WUE response to salinity is very similar to the yield response to salinity. At optimal soil condition, WUE initially increases with salinity to threshold EC, and then decreases. At suboptimal condition, WUE is initially almost constant then sharply reduces with high salinity levels^14^. Similar results have been confirmed by Bompy *et al*.^15^.

Another factor affected by soil water quantity and quality is yield response factor (Ky), which represents the yield response where soil water uptake is restricted. The Ky factor describes the reduction in relative yield, according to the reduction in ET^16^. Lovelli *et al*.^17^ corroborated validity of Ky as a synthesis parameter, in the quantification of the level of tolerance of crops, in terms of water stress. In the case of safflower, a Ky value below 1 indicates tolerance of water deficiency, with minimal impacts on production and overall stability, in terms of water use efficiency. Thus, the species has the mechanism to adapt to limited irrigation and can offer economic sustainability in such irrigation regimes. Conversely, eggplant with a Ky value exceeding 1 shows sensitivity under conditions that include water stress^17^. These results confirm the interactivity of the Ky and WUE parameters^18^, and thus Ky, as an empirical parameter that requires further research testing, may have a value in the estimation of water scheduling needs of specific crops. Zonta *et al*.^16^ rated factor Ky as 0.53 and 0.82, for irrigation of 70% and 40% ETc, respectively. Similarly, Singh *et al*.^19^ rated Ky as 0.28, 0.98 and 1.02 for cotton yields in semi-arid regions of India, for irrigation levels of 90%, 60% and 50% ETc, respectively.

With an increase in demand for irrigation in many global regions, as well as extensive subsidies and restrictive regulations, leading to greater stress on freshwater resources^20^, increases in WUE and reductions in Ky will be required to maintain maximum agricultural productivity with minimum water use^21^. Hence, estimations of WUE and Ky values of specific crops, at a variety of levels of soil salinity and matric potential, are useful for the specification of ideal soil salinity and soil water levels, in terms of available water sources (qualitative and quantitative). Based on these results, practical solutions can be proposed for optimal management, such that crop yields and WUE reach the highest possible levels, without soil salinity increases. Despite the importance of this issue in terms of global sustainability, the effects of soil moisture and salinity interactions on Ky, WUE and WU have rarely been investigated for wet soil (near-saturated to the FC). The purposes of this study are: i) to evaluate the interaction effects of soil aeration and salinity on Ky, WUE, and WU, at FC and higher moisture contents, ii) to investigate the relationship between T and root respiration (Rr) of bean and wheat under salinity and waterlogging, and iii) to estimate critical aeration porosity, according to mentioned parameters and to investigate the accuracy of the hypothesis ‘critical aeration porosity of 10% is not representative for all soils and plants’.

## Materials and Methods

The experiments were undertaken in a research greenhouse (20 ± 5 °C and relative air humidity between 55–80%), using a completely randomized factorial design with three replicates.

### Soil properties

Clay loam and sandy loam soils were selected from 0–0.3 m layers of non-saline, agricultural lands in Zanjan, Iran. Sand particles percentage was measured by a sieve series with diameters of 0.05–2 mm and percentage of silt and clay particles were evaluated based on the Stokes law and hydrometer method^22^. The soils were sampled using cylinders with 100 cm^3^ volume and then soil water characteristics curve (SWCC) was measured by hanging water column at −0.1 to −15 kPa matric potentials (h), using a pressure plate at −33 to −100 kPa matric potentials and by pressure membrane at matric potentials of −150 to −1500 kPa^2^. The van Genuchten-Mualem (VG-M) model was fitted to the SWCC data (θs, α, n) with the RETC program.

Soil electrical conductivity (EC) and reaction (pH) were specified in saturation extract of soils by EC meter (Jenway 4510) and pH meter (Jenway 3505), respectively^23^. The soils were oxidized with sulfuric acid and their nitrogen were measured by Kjeldahl device^24^. Also soils phosphor was determined using Olsen method and Spectrophotometer device (UV/VIS Perkin elmetr- lambada 25-USA)^25^. Finally soils potassium was assessed by ammonium acetate and Flame photometer device (Jenway, PFP-7, UK).

### Plant materials

The soils were collected with an initial field bulk density into plastic pots (0.27 m height and 0.26 m diameter) and an opaque, polyethylene closed chamber, with cross section of 36 × 10^−4^ m^2^ and volume of 5 × 10^−4^ m^3^, was set in each pot^26^. The closed chamber system is a technique which measures the soil respiration rate^27^ (greater detail can be found in Mohammadi *et al*.^26^. A chemical fertilizer solution was added to the soil in each pot, according to soil analysis (Table 1), nutrients leaching and plant type, to enhance growth through the stages of planting, flowering and grain filling^28^ (Table 2). Foliar sprays of micro and macro elements (N = 110 mgl^−1^, P_2_O_5_ = 40 mgl^−1^, K_2_O_5_ = 80 mgl^−1^, Mg = 0.5 mgl^−1^, Mn = 1 mgl^−1^, Cu = 0.2 mgl^−1^, Zn = 3 mgl^−1^, B = 0.2 mgl^−1^, Fe = 2 mgl^−1^, Mo = 0.03 mgl^−1^) were applied during the growth season, for frequently leached pots. Bean (*Phaseoulus vulgaris* cv. COS16) and wheat (*Triticum aestivum* cv. Mahdavi), were planted in the pots. Once the plants had established, eight wheat and four bean plants were subjected to the experimental treatments (soil moisture and salinity). For blank treatment of root respiration (as microbial respiration, Mr) and transpiration experiments, forty pots were prepared without any plants^26^.

**Table 1:** Physical and chemical properties of the studied soils.

Texture	Sand (0.5 × 10^−4^–20 × 10^−4^ m) %	Clay% (<0.02 × 10^−4^ m) %	θs	α	n	BD (Mg m^−3^)	pH	ECe (dSm^−1^)	N (%)	P (mgkg^−1^)	K (mgkg^−1^)
Sandy loam	71	14	0.436	0.38	1.577	1.50	7.71	1.1	1.1	7	432
Clay loam	37	30	0.524	0.72	1.351	1.30	7.72	1.5	1.0	14.2	220

OC = organic carbon and BD = bulk density.

**Table 2:** Details on the fertilizers used.

Urea (Nitrogen fertilizer) (gr/pot)	Potassium sulphate (Potassium fertilizer) (gr/pot)				Ammonium Phosphate (Phosphorus fertilizer) (gr/pot)			
SL and CL	SL		CL		CL		SL	
Wheat and Bean	Wheat	Bean	Wheat	Bean	Wheat	Bean	Wheat	Bean
8.5–17	4.7–12.3	11.15	7.36–14.72	14.72	7.41–14.83	11.13	3.70–7.41	7.41

### Salinity treatments

The irrigation water solutions with salinity levels of 0.7, 2, 4, 6 and 8 dSm^−1^ for bean, and 2, 4, 8, 16 and 20 dSm^−1^ for wheat, were prepared using CaCl_2_ and NaCl (3:1). The salinity treatments levels were determined, according to the FAO report on the ranges of bean and wheat tolerance to salinity^29^.

### Moisture treatments

The −2, −6, −10 and −33 kPa matric potentials were applied as soil moisture treatments for both plants. The matric potentials were conducted using the negative pressure water circulation technique^26,30^. This method authorized us to regulate levels of salinity with high drainage volumes. At treatments of high matric potential (h ≥ −10 kPa), an intensive leaching rate was applied to avoid solute accumulation. At treatments of lower matric potential (h < − 10 kPa), levels of soil salinity were adjusted before seed sowing and de-ionized water was used for irrigation without drainage. Throughout the experiment, the quantities of irrigation and drainage water were recorded accurately for each pot.

$$(2\,{\rm{soils}}\times 2\,{\rm{plants}}\times 5\,{\rm{salinity}}\,{\rm{levels}}\times 4\,{\rm{matric}}\,{\rm{potential}}\,{\rm{levels}}\times 3\,{\rm{replicates}})+(40\,{\rm{blanks}})=280\,{\rm{pots}}$$

### Measurement of root respiration rate

CO_2_ accumulation in the closed chamber, per 0.5 hour (Soil respiration, Sr), was measured^31^, using Lambada device (Lambada, U. K. ADC Company) every 3 days, for 45 days after the plant establishment stage. Root respiration rate, Rr, (μ mol m^−3^ s^−1^) was estimated by^26^:

1 $$Rr=Sr-Mr$$

### Calculation of water use, water use efficiency and yield response factor

At the end of the plant growth (~4 months) after grain filling, shoots (shoot material and grain) and roots were harvested separately. All samples were oven-dried at 70 °C for at least 72 hours, after which they were weighed and the biomass of shoots (Bs) and roots (Br) were measured.

Volume of water use (WU, m^3^/pot) or ET (evapotranspiration) during the experiment was calculated as follows:

2 $$WU=Wi-Wd$$

where, W_i_ and W_d_ are the total amounts of irrigation and drainage water in the planted pots, respectively.

Evaporation (E) was estimated by Eq. (3)

3 $${\rm{E}}={\rm{Wi}}({\rm{b}})-{\rm{Wd}}({\rm{b}})$$

where, Wi(b) and Wd(b) are the total amounts of irrigation and drainage water in non-planted pots, respectively.

Transpiration (T) was calculated as follows:

4 $${\rm{T}}={\rm{WU}}-{\rm{E}}$$

WUE (kgm^−3^) of shoot was obtained by mass of shoot biomass divided by volume of water use^32^:

5 $$WUE=Bs/WU$$

Finally Ky was calculated by^32^:

6 $$(1-Bs/B{s}_{max})={K}_{y}(1-ET/E{T}_{max})$$

where Bs and Bs_max_ are the actual biomass and maximum biomass of shoots, and ET and ET_max_ are the evapotranspiration and maximum evapotranspiration, respectively.

The statistical analyses were performed using SAS 9.1.3 statistics software. The effect of the treatments and the interactive effects between them were investigated using the Duncan multiple range test.

## Results

Physical and chemical properties of the soils used in the greenhouse experiment are given in Table 1.

The variance analysis table shows that soil matric potential has a significant influence on bean and wheat Bs, WU, WUE and Rr (p < 0.01). The statistical analysis also confirms the influence of soil salinity on bean and wheat Bs, WU and WUE (p < 0.01). The effect of salinity on wheat Rr (p < 0.01) is more significant than on bean (p < 0.05). The interaction effects of soil salinity and matric potential on all parameters of wheat, and WU and Rr of bean, are significant (p < 0.01). However, there was no effect on bean Bs and WUE (Table 3).

**Table 3:** Variance analysis of the effects of soil salinity (EC) and matric potential on the shoot biomass (kg/plant), Bs, water use (m^3^/pot), WU, and water use efficiency (kgm^−3^), WUE, of bean and wheat.

Dependent Variable	Freedom degree	Mean square					
		Bs		WU		WUE	
		wheat	bean	wheat	Bean	wheat	Bean
Potential	3	0.85 × 10^−5**^	5.09 × 10^−5**^	0.11 × 10^−3**^	0.12 × 10^−3**^	1.46^**^	5.09^**^
EC	4	3.31 × 10^−5**^	4.46 × 10^−5**^	0.07 × 10^−3**^	0.06 × 10^−3**^	8.24^**^	1.80^**^
EC $\times$ potential	12	0.22 × 10^−5**^	0.15 × 10^−5^	0.005 × 10^3−**^	0.003 × 10^−3**^	0.76^**^	0.19
Error		0.01 × 10^−5^	0.15 × 10^−5^	0.0005 × 10^−3^	0.0007 × 10^−3^	0.10	0.22
CV		13.25	29.045	6.96	9.81	13.98	25.44

^**^ and ^*^ mean significant effects at 0.01 and 0.05 levels of probability, respectively.

### Biomass of shoot

**Figure 1:**
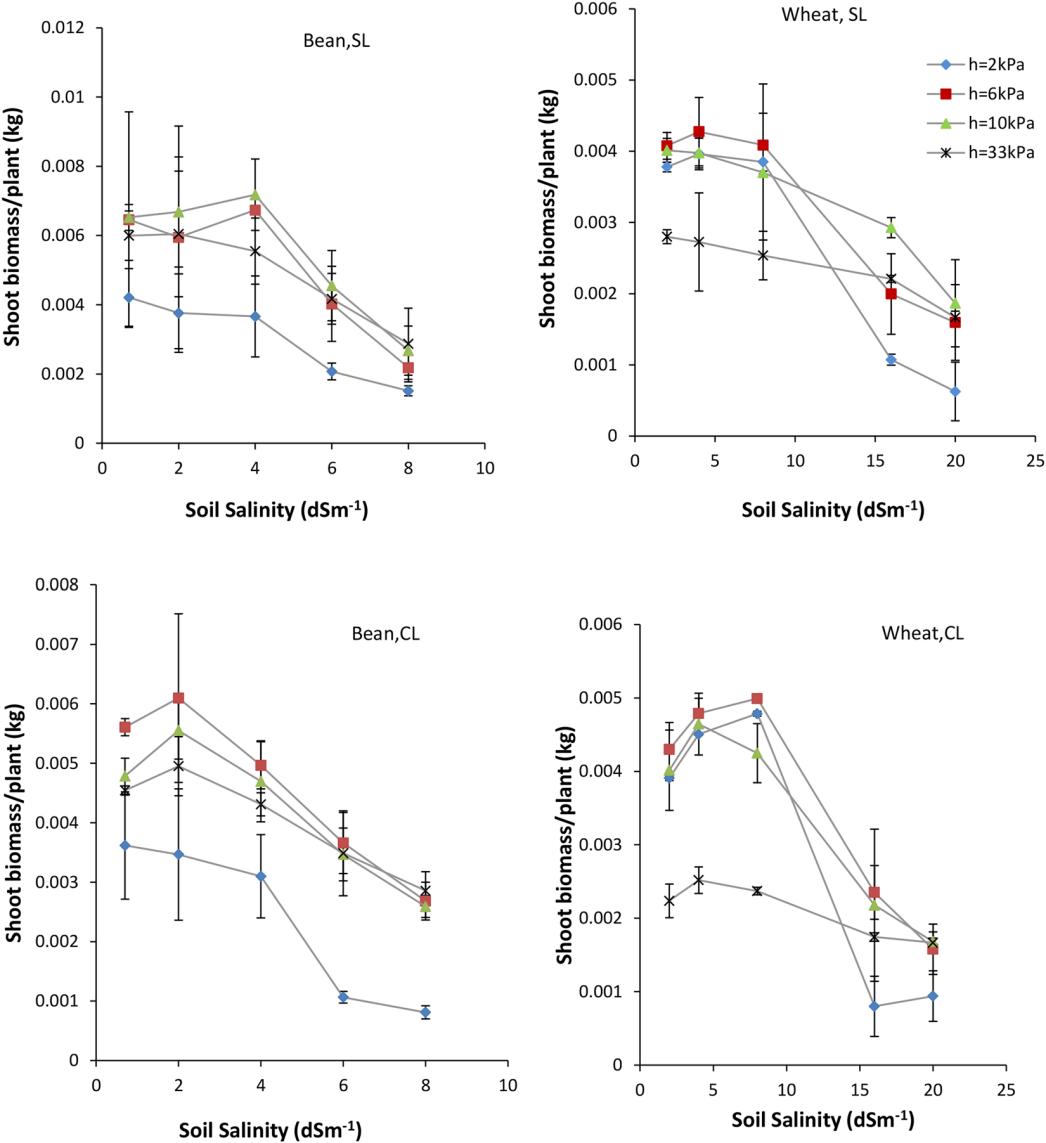
Shoot biomass of wheat and bean as a function of soil salinity under different soil salinities (EC) in study soils. Error bars show one standard deviation around the mean.

The variations of Bs (kg/plant), as a function of soil salinity for the planted wheat and bean, are shown in Fig. 1. The results show that at −2 kPa matric potential, wheat Bs is lower than −6 kPa potential under EC ≤ 8 dSm^−1^ and is lower than other potentials (h < − 2 kPa) under EC > 8 dSm^−1^. The bean Bs is the minimum at −2 kPa potential, under all salinity levels, in sandy loam and clay loam soils. As the soil matric potential decreases slightly, the wheat and bean Bs increase, reaching maximum value at −6 to −10 kPa potentials in both soils. At matric potentials lower than −10 kPa, and under salinities lower than 8 dsm^−1^ for wheat and 4 dsm^−1^ for bean, Bs decreases with reduction of potential (especially for wheat), while under higher salinities it remains nearly constant (Fig. 1).

Salinities lower than 8 dSm^−1^ for wheat, and 4 dSm^−1^ for bean, do not significantly affect Bs (Table 4). Under higher salinities, the Bs of both plants decrease in sandy loam and clay loam soils. The highest effect of salinity on wheat and bean Bs is at −2 kPa potential. As matric potential decreases, the salinity effect reduces and at −33 kPa potential is minimized in sandy loam and clay loam soils (Fig. 1). The minimum Bs of both plants was observed at −2 kPa potential under EC = 20 dsm^−1^ for wheat, and EC = 8 dsm^−1^ for bean, in clay soil (Fig. 1).

**Table 4:** Mean comparisons of Bs (kg/plant), WU (m^3^/pot) and WUE (k gm^3^) for wheat and bean as affected by soil matric potential (h) and salinity (EC).

Treatment	Levels	Wheat			Levals	Bean		
		Bs	WU	WUE		Bs	WU	WUE
h	−2	0.0028b	0.0102c	2.08c	−2	0.0023b	0.0070c	1.41c
	−6	0.0034a	0.0116a	2.28b	−6	0.0048a	0.0110a	1.71b
	−10	0.0033a	0.0112b	2.44ab	−10	0.0052a	0.0104b	1.86b
	−33	0.0022b	0.0073d	2.60a	−33	0.0044a	0.0074c	2.40a
EC	2	0.0036a	0.0115a	2.58a	0.7	0.0054a	0.0106a	1.95ab
	4	0.0039a	0.0114ab	2.83a	2	0.0052a	0.0103a	2.08a
	8	0.0038a	0.0110b	2.87a	4	0.0048a	0.0095b	2.06a
	16	0.0019b	0.0086c	1.89b	6	0.0033b	0.0079c	1.70bc
	20	0.0014c	0.0078d	1.57c	8	0.0023c	0.0066d	1.44c

*In each column and for each group, means with different letters are significantly different at P < 0.05.

Comparing sandy loam and clay loam soils shows that the variations of Bs in terms of salinity and matric potential are almost identical in both soils, indicating that soil texture does not affect Bs dramatically.

The results show that reduction of wheat and bean shoot biomass with increasing of one salinity unit (b) is the maximum value at −2 kPa potential, especially for wheat in sandy loam and clay loam soils. As soil matric potential decreases from −2 to −33 kPa, b decreases, with lowest values in sandy loam and clay loam soils obtained at −33 kPa potential (Fig. 1).

### Volume of water use

**Figure 2:**
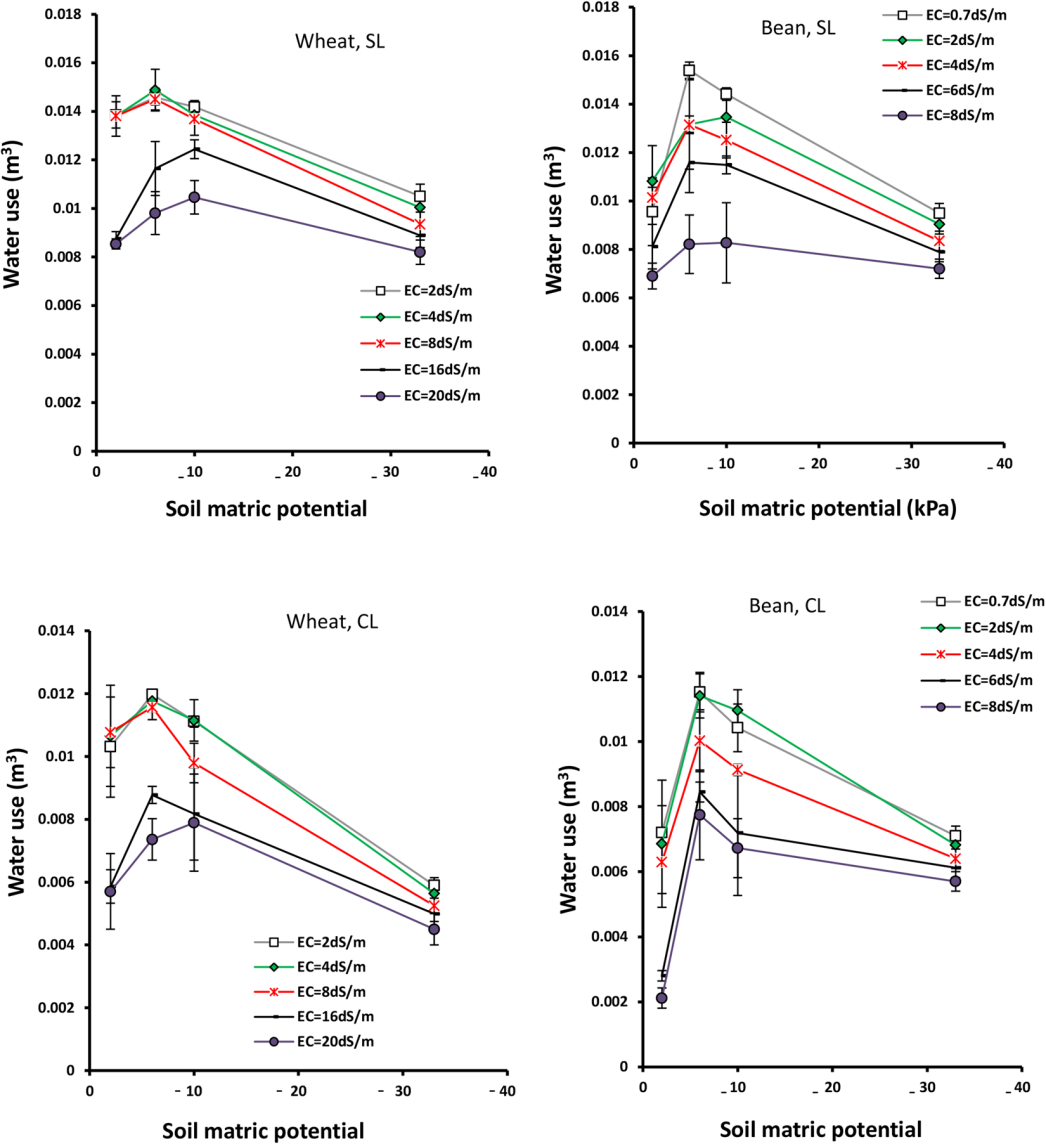
Volume of water use of wheat and bean as a function of matric potential under different soil salinities (EC), in sandy loam and clay loam soils. Error bars show one standard deviation around the mean.

The results shows that variations of water use (WU) for wheat and bean, in terms of soil matric potential and salinity, are similar to those for Bs. The wheat WU at −2 kPa matric potential is lower than −6 to −10 kPa potential, and bean WU reaches minimum value at −2 kPa potential, rather than other potentials (h < −2 kPa) in both sandy loam and clay loam soils (Table 4). As matric potential decreases from −2 to −6 or −10 kPa, wheat and bean WU reach their maximum values and then collapse at −33 kPa in sandy loam and clay loam soils (Fig. 2).

Salinities lower than 4 dSm^−1^ for bean and 8 dSm^−1^ for wheat do not seriously affect WU (Table 4). Under higher salinities, wheat and bean WU reduces significantly, particularly at −2 kPa potential, in both soils (Table 5). The minimum WU is 0.004 m^3^/pot under EC = 20 dSm^−1^ and −33 kPa potential for wheat, and 0.002 m^3^/pot under EC = 8 dSm^−1^ and −2 kPa potential for bean, in clay loam soil (Fig. 2).

**Table 5:** Mean comparisons of the interactive effects of matric potential (h) and salinity (EC) on Bs (kg/plant), WU (m^3^/pot) and WUE (kgm^3^) for wheat and bean.

Treatment	Wheat				Treatment		Bean
EC	h	Bs	WU	WUE	EC	h	WU
2	−2	0.0038b	0.0120c	2.63a–c	0.7	−2	0.0083f-h
4	−2	0.0042ab	0.0122bc	2.86a	2	−2	0.0088fg
8	−2	0.0043ab	0.0122bc	2.98a	4	−2	0.0082f-h
16	−2	0.0009hi	0.0073 g	1.02e	6	−2	0.0054ki
20	−2	0.0007i	0.0071gh	0.93e	8	−2	0.0045i
2	−6	0.0041ab	0.0133a	2.55a–c	0.7	−6	0.0134a
4	−6	0.0045a	0.0132a	2.77ab	2	−6	0.0122b
8	−6	0.0045a	0.0130ab	2.82ab	4	−6	0.0115bc
16	−6	0.0021c-e	0.0102d	1.75d	6	−6	0.0100de
20	−6	0.0015e	0.0085ef	1.49d	8	−6	0.0079gi
2	−10	0.0040ab	0.0126a-c	2.57a–c	0.7	−10	0.0124b
4	−10	0.0043ab	0.0124a-c	2.82ab	2	−10	0.0122b
8	−10	0.0039b	0.0117c	2.81ab	4	−10	0.0108cd
16	−10	0.0025c	0.0103d	2.41bc	6	−10	0.0093ef
20	−10	0.0017e–g	0.0091ce	1.58d	8	−10	0.0075h-j
2	−33	0.0025c	0.0082 f	2.58a–c	0.7	−33	0.0083f-h
4	−33	0.0026c	0.0078fg	2.87a	2	−33	0.0079g-i
8	−33	0.0024cd	0.0073 g	2.89a	4	−33	0.0073h-j
16	−33	0.0019d-f	0.0069gh	2.39bc	6	−33	0.0070ij
20	−33	0.0013gh	0.00063 h	2.28c	8	−33	0.0064jk

In each group, means with at least one similar letter are not significantly different at P < 0.05 (Duncan multiple range test).

Figure 2 shows that wheat WU is higher than bean under all salinities, particularly under anoxia condition (−2 kPa potential).

### Water use efficiency

**Figure 3:**
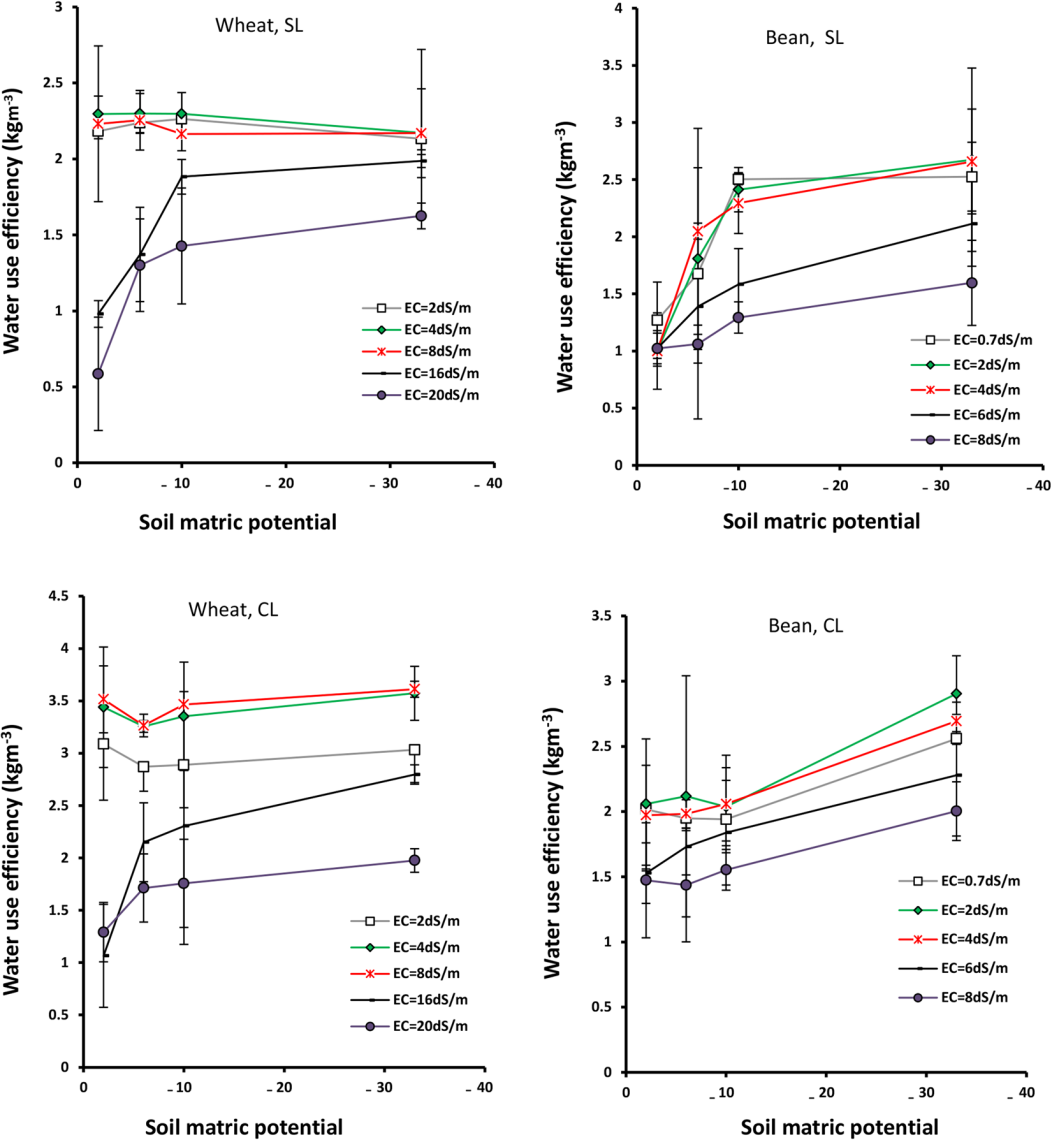
Water use efficiency of wheat and bean as a function of soil matric potential (kPa) under different salinities (EC), in sandy loam and clay loam soils. Error bars show one standard deviation around the mean.

Figure 3 shows that the wheat WUE remains almost constant with reductions of matric potential under EC = 2–8 dSm^−1^, while when EC > 8 dSm^−1^, it increases with reduction of matric potential in sandy loam and clay loam soils. The bean WUE also increases with reductions of matric potential under all salinities and in both soils (Fig. 3). The maximum WUE of wheat and bean are 3.7 and 2.9 kgm^−3^, respectively at −33 kPa potential, while their minimum values are 1.1 and 1.5 kgm^−3^, respectively at −2 kPa potential, in clay loam soil (Fig. 3). At −33 kPa potential, wheat WUE is 20% and bean WUE is 41% greater than their WUE at −2 kPa potential (Table 4).

Salinities lower than 8 dSm^−1^ for wheat, and 4 dSm^−1^ for bean, do not significantly affect WUE (Table 4). Under higher salinities, WUE decreases severely with a minimum value under EC = 20 dSm^−1^ for wheat, and EC = 8 dSm^−1^ for bean, at all potentials and in both soils. Under these salinities, wheat WUE drops by 39% (rather than EC = 2 dSm^−1^) and bean by 26% (rather than EC = 0.7 dSm^−1^) (Table 4). The effect of salinity on wheat WUE is more pronounced at higher potentials and minimizes at −33 kPa potential (Fig. 3).

Figure 3 shows that the variations of WUE, in terms of salinity, are almost the same in both soils, as well as the variations of WUE according to matric potential. The results reveal that wheat WUE exceeds bean WUE in sandy loam and clay loam soils.

**Figure 4:**
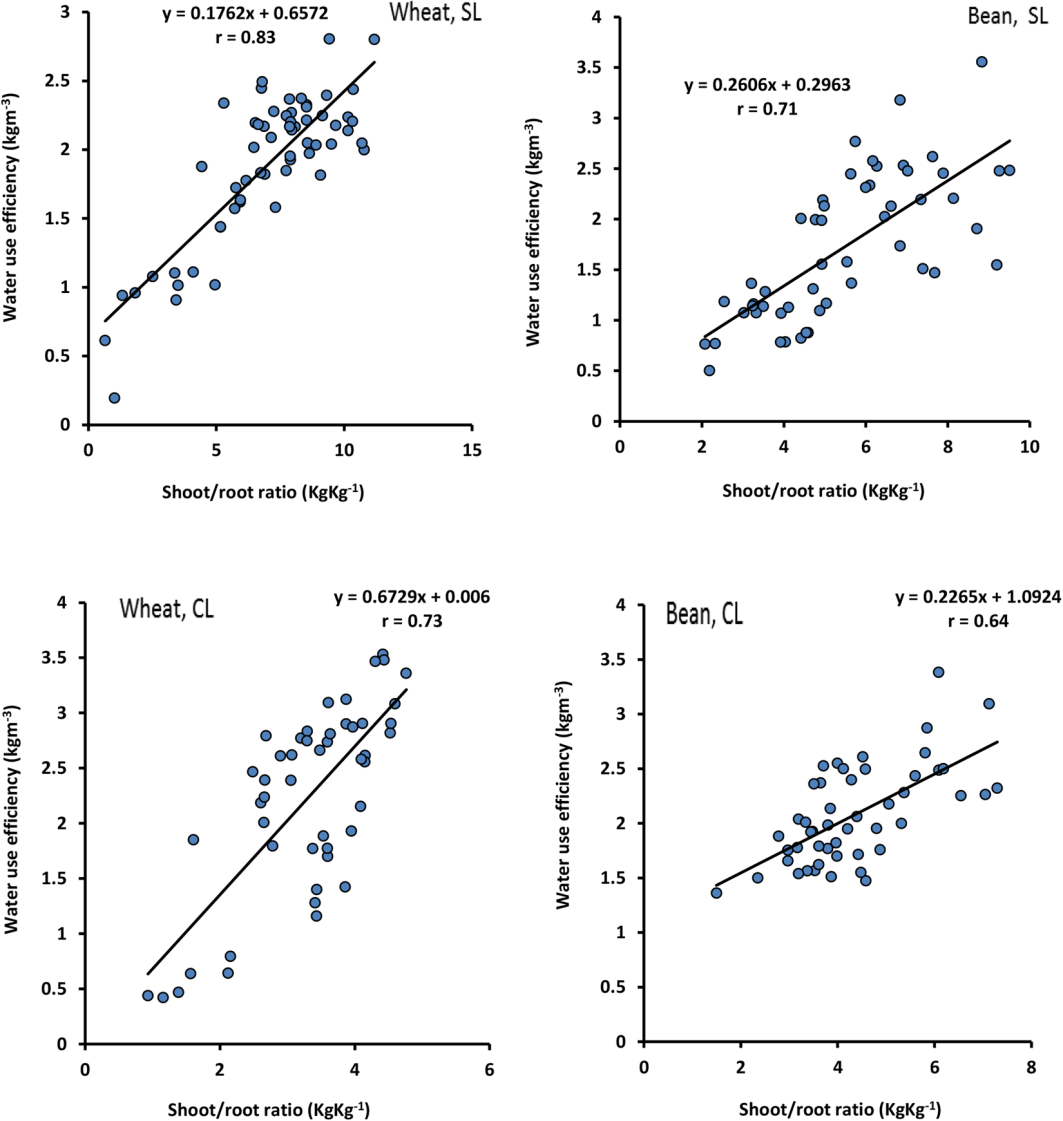
Water use efficiency of wheat and bean as a function of shoot/root ratio, in sandy loam and clay loam soils.

Figure 4 shows a significant relationship between WUE and the shoot/root ratio for wheat and bean, and the strong correlation coefficient indicates the considerable effect of the shoot/root ratio on WUE. The wheat correlation coefficient (0.83–0.73) is higher than bean (0.71–0.64) in sandy loam and clay loam soils. The wheat slope of regression line in sandy loam soil (0.17) is lower than in clay loam soil (0.67), while for bean it is the same in both soils (0.2).

### Yield response factor

**Figure 5:**
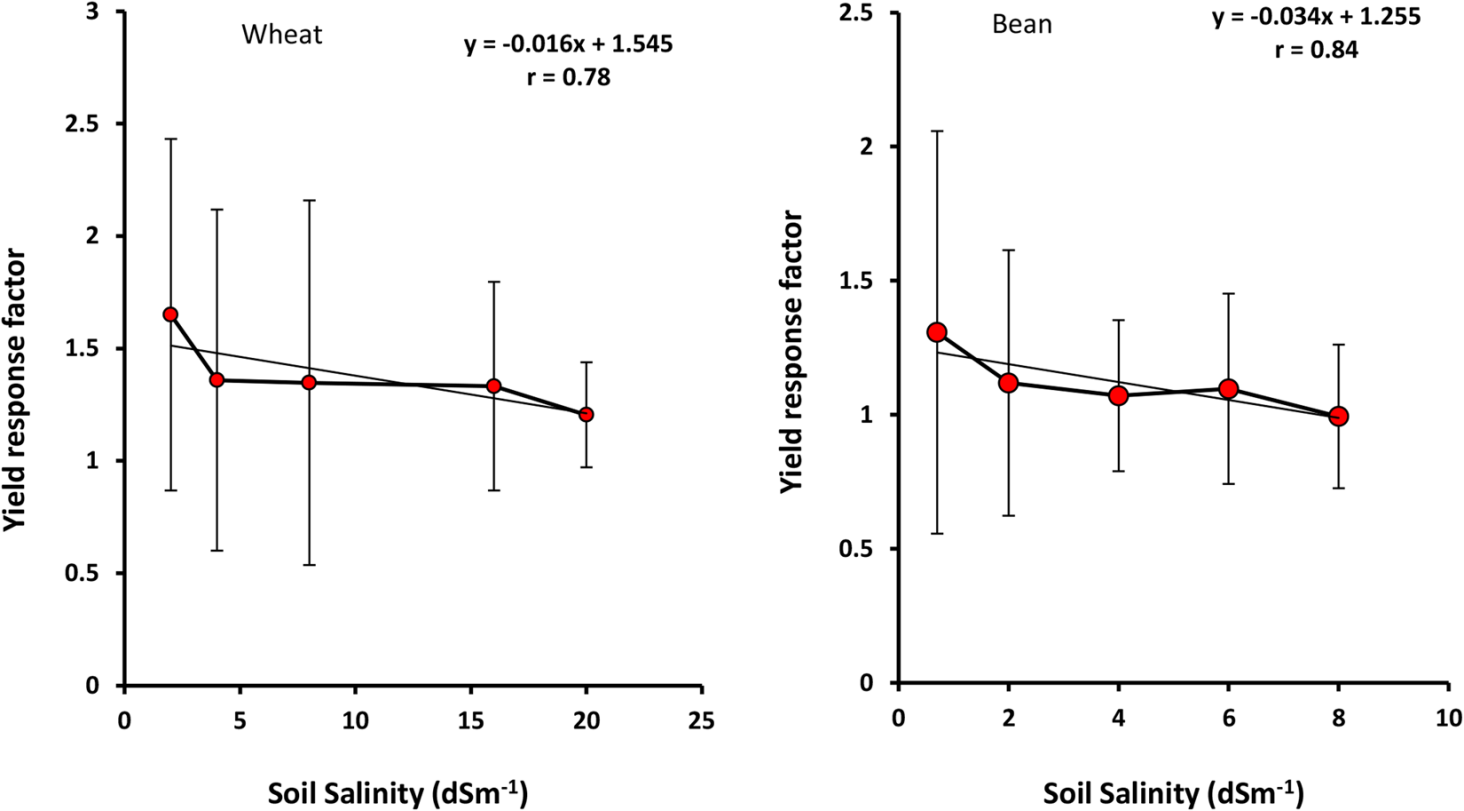
Yield response factor of wheat and bean as a function of soil salinity (EC). Error bars show one standard deviation around the mean.

**Figure 6:**
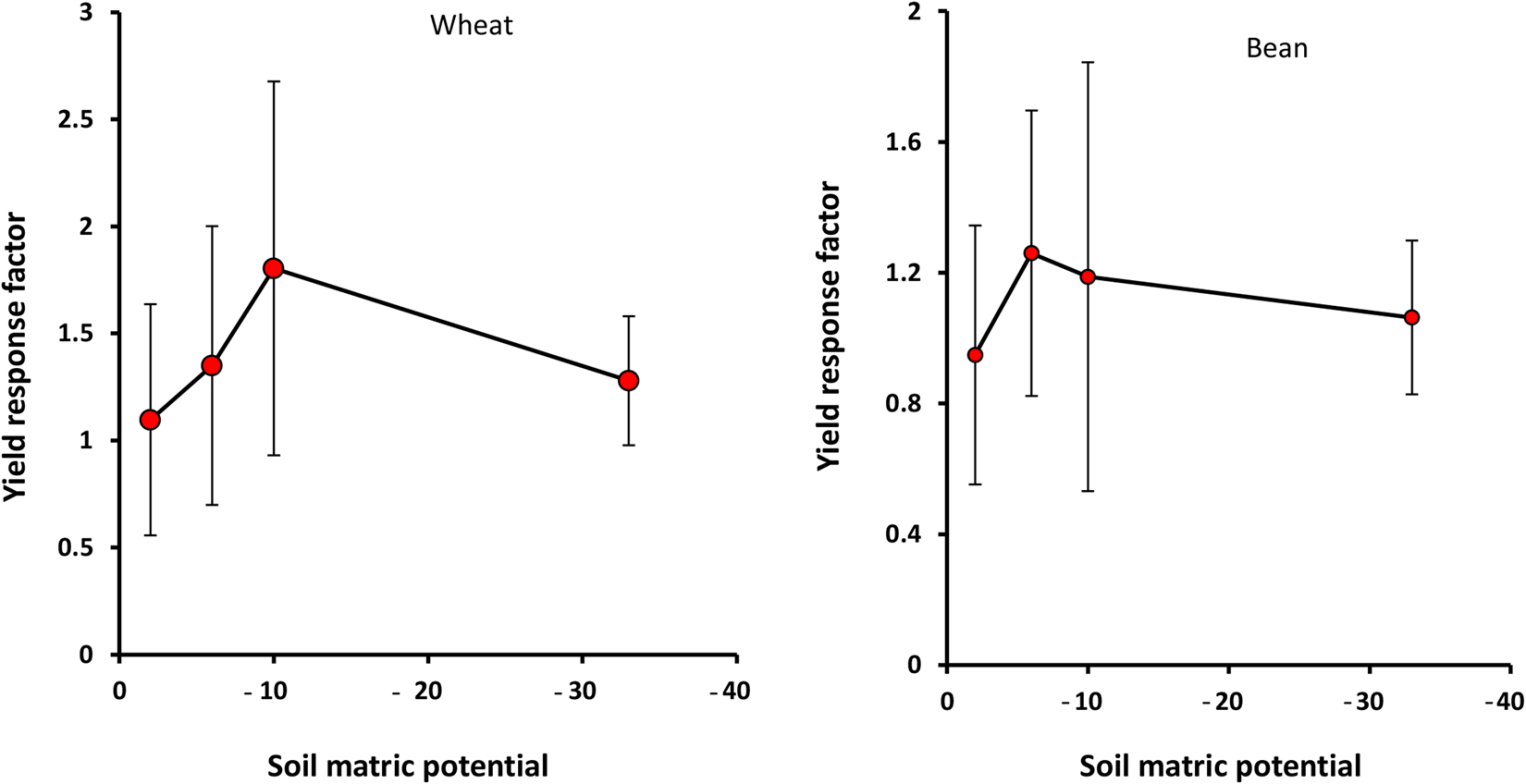
Yield response factor of wheat and bean as a function of soil matric potential (kPa). Error bars show one standard deviation around the mean.

Since Ky is crop specific and is not influenced by soil type, Ky (for both soils) as a function of soil salinity and matric potential is given in Figs 5 and 6, respectively.

The Ky is the maximum value and equals 1.65 under EC = 2 dSm^−1^ for wheat and 1.30 under EC = 0.7 dSm^−1^ for bean. Wheat and bean Ky decrease with salinity and the high regression coefficient (0.78–0.84, respectively) demonstrates the significant effect of salinity on both crops. Figure 5 shows that bean Ky reduces more than wheat, in terms of salinity, and its slope of regression line (3.4%) is more than that of wheat (1.6%). Further, wheat Ky values are higher than bean under all salinities (Fig. 5).

The results reveal that under waterlogging (−2 kPa potential), wheat and bean Ky values are minimum and equal 1.1 and 0.9, respectively. As matric potential decreases, Ky increases and reaches a maximum value at −10 kPa potential for wheat (1.8) and at −6 kPa potential for bean (1.3) (Fig. 6). The increment of bean Ky with reduction of potential is greater than in the case of wheat. At lower potentials (h < −10 kPa for wheat and h < −6 kPa for bean), Ky decreases significantly, especially for wheat (Fig. 6).

### Relationships between rate of root respiration and transpiration

**Figure 7:**
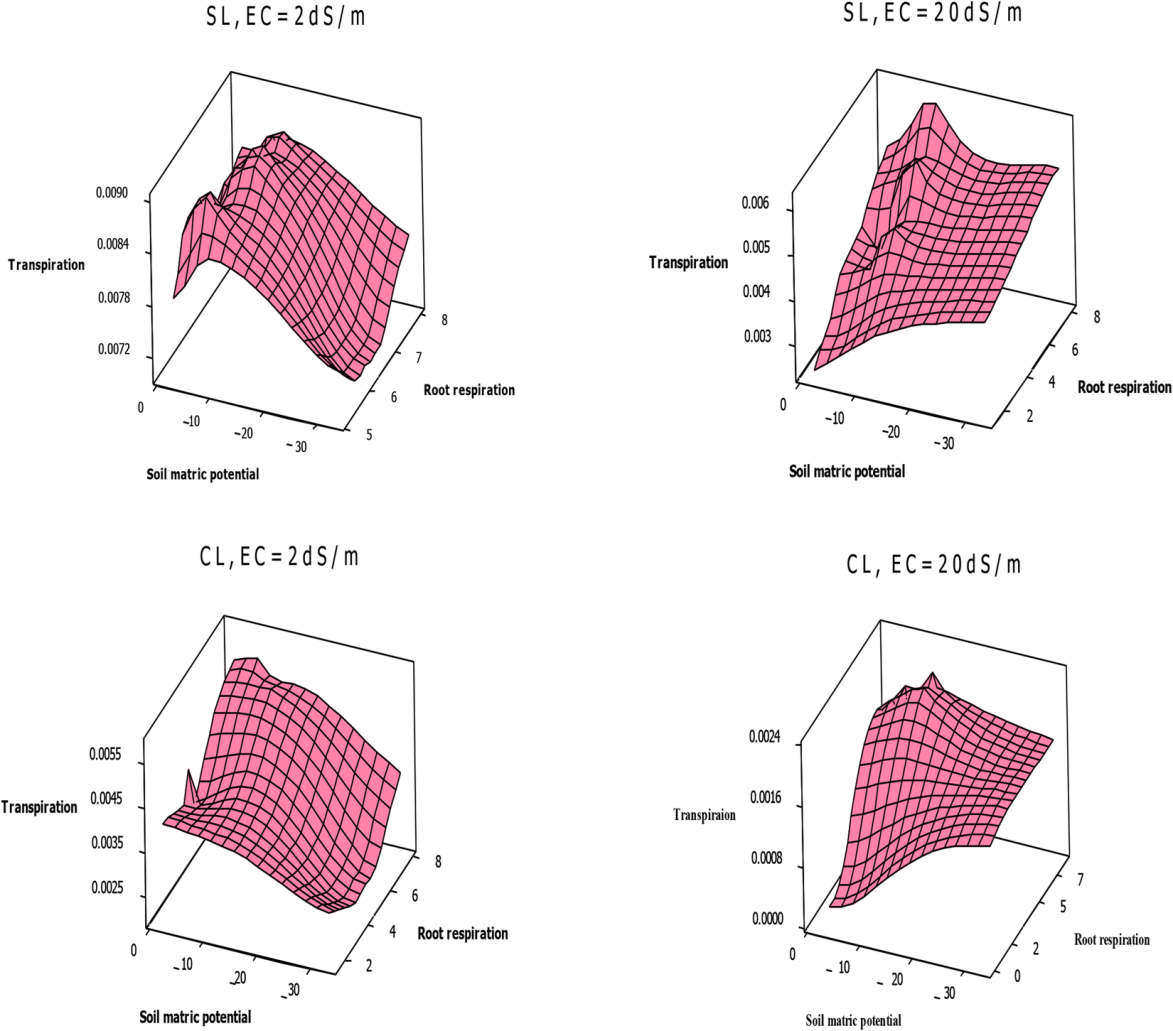
Wheat transpiration rate (m^3^/pot) as a function of root respiration rate (μ mol m^−3^ s^−1^) and soil matric potential (kPa), under EC = 2 dSm^−1^ and 20 dSm^−1^, in sandy loam and clay loam soils.

**Figure 8:**
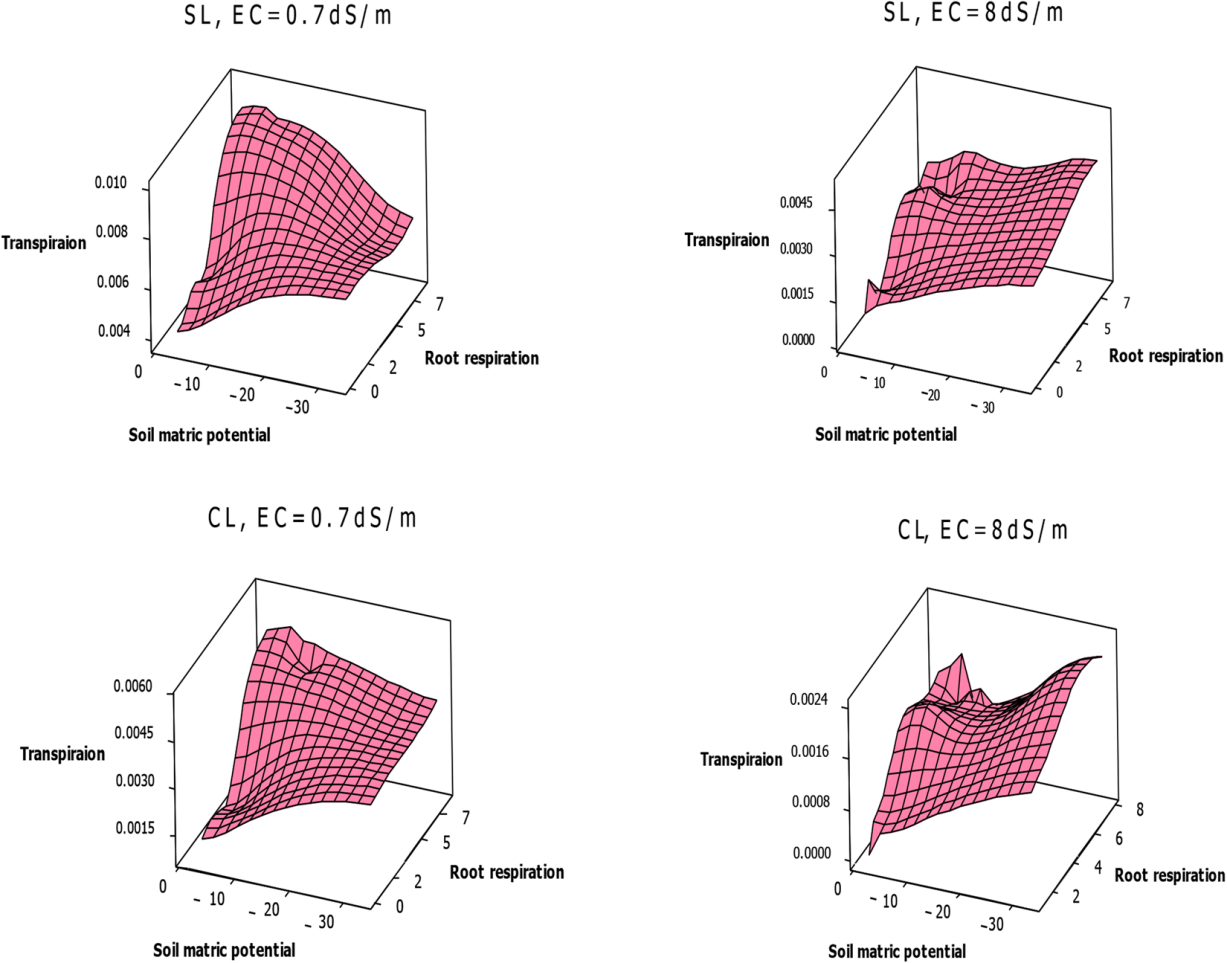
Bean transpiration rate (m^3^/pot) as a function of root respiration rate (μ mol m^−3^ s^−1^) and soil matric potential (kPa), under EC = 0.7 dSm^−1^ and 8 dSm^−1^, in sandy loam and clay loam soils.

The variations of transpiration (T) as a function of root respiration (Rr, μ mol m^−3^ s^−1^) and soil matric potential, under salinities lower and higher than threshold, for wheat (EC = 2 and 20 dSm^−1^) and bean (EC = 0.7 and 8 dSm^−1^) are shown in Figs 7 and 8, respectively. The results show that T is affected by soil potential and Rr.

In sandy loam and clay loam soils, under salinities lower than threshold, wheat T decreases with decreasing potential and Rr, reaching a minimum value at −33 kPa. Under higher salinities, wheat T lowest value is at −2 kPa potential (Fig. 7). Bean T is minimum at −2 kPa potential, and increases with decreasing potential and increasing Rr under all salinity levels (Fig. 8). A comparison of the two plants illustrates that wheat Rr and T values exceed those for bean in both soils (Figs 7 and 8).

## Discussion

### The effects of soil matric potential

This study has shown that Bs, WU, WUE, Rr and Ky are strongly influenced by soil aeration and decrease at −2 kPa potential (Figs 1 and 2). At high matric potential (−2 kPa), or high soil water content, pores are blocked by water and oxygen deficit results in stomata closure and restricted water uptake. Subsequently, the decline of the photosynthetic rate and root permeability lead to decreased growth and impeded plant activities, such as transpiration and root respiration^33^. Wheat adapts somewhat to soil anoxia^34^ by developing aerenchyma tissues^35^ and adventitious roots^36^. Thus, wheat WU and WUE values are higher than those for bean under anoxia condition (−2 kPa potential), confirming the high tolerance of wheat to stresses (Figs 2 and 3).

As the soil matric potential decreases (to −6 or −10 kPa), soil aeration and root uptake of water and nutrients improves, such that wheat and bean Bs and WU increase, reaching maximum values at −6 to −10 kPa potential, in both soils (Figs 1 and 2). Consequently, Ky also increases to a maximum value at −10 kPa potential for wheat, and at −6 kPa potential for bean (Fig. 6). It seems that these matric potentials correspon d with optimum soil moisture conditions, which can be considered as the critical aeration porosity (0.18–0.16 m^3^m^−3^ in sandy loam and clay loam, respectively), in which there is equilibrium between the quantities of water and air, resulting in both reasonable respiration and water uptake. This result supports the hypothesis that 10% critical aeration porosity is not sufficient for all plants and soils.

Wheat and bean WUE also increases with aeration improvement (Fig. 3), due to the fact that increases of Bs (numerator) with decreasing potential exceed WU (denominator), and their ratio which is defined as WUE, thus increases. The increase of WUE with reduction of soil potential has been observed by many researchers (e.g. see Ghamarnia *et al*.^37^). Markandya *et al*. concluded that flood irrigation WUE is nearly 35%, and equivalent to half and one third of the sprinkler and drip irrigation systems^38^, respectively. Flood irrigation can result in decreasing water uptake and inefficient use of fertilizer, ultimately decreasing WUE^39^. The flooding effect on WUE depends on the type of plant. Fahong *et al*.^39^ showed that wheat WUE increased 30% by changing from flood to furrow irrigation, while rice WUE under field capacity is lower than that under flooding^40^. At potentials lower than −6 to −10 kPa, because of deviation from optimum moisture condition and decreasing of soil non-limiting water, hydraulic conductivity and salt mass flow, water uptake by root reduces, leading to decreased Bs, WU (or evapotranspiration) and Ky (Figs 1, 2 and 6). The WU decline can be also attributed to a decrease in shoots and surface leaves that affect wheat and bean transpiration adversely (Fig. 1). Further, at these lower potentials, evaporation of soil water content decreases, strongly affecting WU. This result is comparable with the results observed by Kiani^41^.

### The effects of soil salinity

The results have shown that high salinities (EC > 8 dSm^−1^ for wheat and EC > 4 dSm^−1^ for bean) decrease Bs, WU, WUE and Rr in both plants, and the effects are greater in wheat rather than bean (Figs 1, 2, 3, 7 and 8). In addition, Ky decreases significantly with salinity (Fig. 5). Similar results were obtained for corn (Azizian and Sepaskhah, 2014), while Shabani *et al*.^42^ reported that rapeseed Ky increases with salinity to threshold salinity, and then decreases under higher salinities. Ion toxicity (especially sodium), ion balance disorder and restricted uptake of water and nutrient are the most important reasons for yield reduction, and related factors, under high salinities^43^. This result has been confirmed by many researchers^14,44,45^.

### The interaction effects of soil salinity and matric potential

Oxygen deficit can intensify the effects of salinity stress. This synergic effect results in major decreases of Bs, WU and WUE at high potentials (−2 kPa) under high salinities (Figs 1, 2 and 3). As soil potential decreases from −2 to −33 kPa, the adverse effect of salinity on wheat and bean diminishes, and reduced soil moisture becomes the major controlling factor of plant growth and development (Fig. 1). Based on this result, it can be stated that at the upper limit of the water range (high soil moistures or high matric potentials), salinity affects plant growth significantly, while at the lower limit (low soil moistures or low matric potentials), the effect of salinity is minimal and plant water uptake and growth are barely impacted by matric potential.

Figures 5 and 6 confirm that Ky value is minimum under stress conditions (waterlogging and salinity). Since biomass decline (numerator) by stresses is lower than that of evapotranspiration, or WU (denominator), their ratio (Ky) reduces under waterlogging and salinity. In other words, under suitable environmental conditions (at optimum matric potential and low salinity), plant sensitivity to stresses is very high^42^.

Ky values for bean and wheat are higher than 1 (Ky > 1) for all salinities and matric potentials. These values are reported by FAO as 1.15 for both plants^18^. This indicates that neither plant adapts well to waterlogging and salinity.

Under low salinities, wheat can extend its root system and adapt to anoxia that its Rr increases at −2 kPa potential. Subsequently, due to enhanced water and nutrient uptake, shoots are extended, leading to increased T. Since salinity intensifies waterlogging, under high salinities wheat roots and shoots decrease severely and Rr and T decline to a minimum value at −2 kPa potential (Fig. 7). Bean is very sensitive to waterlogging, so its minimum Rr and T are remain −2 kPa potential, for all salinity levels (Fig. 8).

### The relation between root respiration and transpiration

Figures 7 and 8 show that wheat and bean Rr affects T and that there is a significant relationship between these factors in sandy loam and clay loam soils. With treatments of matric potential and salinity, the wheat and bean T variations are almost similar to those of Rr (Figs 7 and 8). Under stress conditions, shoot and root growth and plant activities such as photosynthesis and root respiration decrease, leading to decreased T^46^. These results confirm that T, and subsequently WUE, can be controlled by Rr regulation. The Rr rate depends on environmental factors and plant type, age and activity^33^. In the case of the two study plants, due to the extensive root system of wheat, its mean Rr (5.79 μ mol m^−3^ s^−1^) is higher than that of bean (5.69 μ mol m^−3^ s^−1^) (Figs 7 and 8). Although their values are closed, this difference is considerable biologically.

### The relation between shoot/root ratio and water use efficiency

WUE increases with shoot/root ratio, indicating a significant relationship between them (Fig. 4). An higher shoot /root ratio causes an increase in transpiration and photosynthesis rate, and consequently WUE improves under irrigated conditions. Ma *et al*.^47^ reported similar results and showed that controlling the root/shoot ratio can affect WUE.

## Conclusions

This study was conducted to investigate variations in water use, water use efficiency and yield response factor in bean and wheat, as a function of soil matric potential and salinity in sandy loam and clay loam soils. The results showed firstly that in wet soil, soil aeration porosity is the principal factor determining these parameters and that secondly, the effects of soil salinity, soil texture and crop type are significant. At high matric potentials, and under waterlogging, the parameters referred to are very low and reach maximum values at optimum matric potentials (−6 to −10 kPa). These matric potentials correspond with aeration porosities of 0.16–0.18m^3^m^−3^. This result demonstrates that a critical aeration porosity of 10% does not hold for different plants and soils. The low to medium salinities do not significantly affect water use, water use efficiency and yield response factor. Conversely, high salinities decrease all parameters significantly, and intensify the effect of oxygen deficit at high potentials. Thus, in the absence of a quality water source, in order to minimize the effect of salinity and improve WUE, it is more expedient to control soil potential in critical aeration porosity (according to soil and plant). Comparing our two study plants indicates the greater ability of wheat as regards water uptake and growth under stress conditions. The results also showed that root respiration affects transpiration, and that a significant relationship exists between these two functions. Thus, by regulating root respiration and transpiration rate, WUE can be effectively controlled^48^.
