# Author Correction: Soil salinity and matric potential interaction on water use, water use efficiency and yield response factor of bean and wheat

**DOI:** 10.1038/s41598-018-31452-z

**Published:** 2018-08-31

**Authors:** Mahnaz Khataar, Mohammad Hossien Mohammadi, Farzin Shabani

**Affiliations:** 1 Department of Soil Science, University of Zanjan, Zanjan, Iran; 2 Department of Soil Science Faculty of Agriculture and Natural Resources, University of Tehran, Karaj, Iran; 3 Ecosystem Management, School of Environmental and Rural Science, University of New England, Armidale, NSW 2351 Australia

Correction to: *Scientific Reports* 10.1038/s41598-018-20968-z, published online 08 February 2018

The original version of this Article contained a typographical error in the spelling of the author Mohammad Hossien Mohammadi, which was incorrectly given as Mohammad Hossien Mohhamadi.

In addition, the original version of this Article contained errors in the Materials and Methods section.

Under the subheading ‘Soil Properties’,

“The soils were sampled using cylinders with 100 cm^3^ volume and then soil water characteristics curve (SWCC) was measured by hanging water column at −0.1 to −15 kPa matric potentials (h), using a pressure plate at −33 to −100 kPa matric potentials and by pressure membrane at matric potentials of −150 to −1500 kPa 26.”

now reads:

“The soils were sampled using cylinders with 100 cm^3^ volume and then soil water characteristics curve (SWCC) was measured by hanging water column at −0.1 to −15 kPa matric potentials (h), using a pressure plate at −33 to −100 kPa matric potentials and by pressure membrane at matric potentials of −150 to −1500 kPa^2^.”

Under the subheading ‘Moisture treatments’,

“The matric potentials were conducted using the negative pressure water circulation technique^30^”

now reads:

“The matric potentials were conducted using the negative pressure water circulation technique^26,30^”

Furthermore, the legend of Figure 3,

“Water use efficiency of wheat and bean as a function of soil matric potential under different salinities (EC), in sandy loam and clay loam soils. Error bars show one standard deviation around the mean.”

now reads:

“Water use efficiency of wheat and bean as a function of soil matric potential (kPa) under different salinities (EC), in sandy loam and clay loam soils. Error bars show one standard deviation around the mean.”

Finally, the legend of Figure 6,

“Yield response factor of wheat and bean as a function of soil matric potential (EC). Error bars show one standard deviation around the mean.”

now reads:

“Yield response factor of wheat and bean as a function of soil matric potential (kPa). Error bars show one standard deviation around the mean.”

These errors have now been corrected in the PDF and HTML versions of the Article.

